# Time resolved measure of coronary sinus flow following regadenoson administration

**DOI:** 10.1186/1532-429X-13-S1-O74

**Published:** 2011-02-02

**Authors:** O Julian Booker, Patricia Bandettini, Peter Kellman, Joel Wilson, Steve Leung, Sujethra Vasu, Sujata Shanbhag, Jennifer Henry, Tracy Lowrey, Christine Mancini, Andrew E Arai

**Affiliations:** 1 National Heart, Lung, and Blood Institute, Bethesda, MD, USA

## Objective

To use velocity encoded phase contrast MRI to determine timing of peak myocardial blood flow to establish when CMR stress perfusion imaging should be performed after injection of regadenoson.

## Background

Regadenoson is a selective A2A adenosine receptor agonist recently FDA approved for stress testing. The package insert recommends administration of the radionuclide imaging agent 20 seconds after bolus. Optimal timing of CMR first-pass perfusion imaging has not been established.

## Methods

CMR was performed on eighteen volunteers with 10-year Framingham risk scores <1% (15 m, 23 ± 7 years) using a 1.5 T Siemens Espree. Serial measures of coronary sinus (CS) and cardiac output (CO) were made using a velocity encoded phase contrast sequence.

## Results

**Figure 1:**
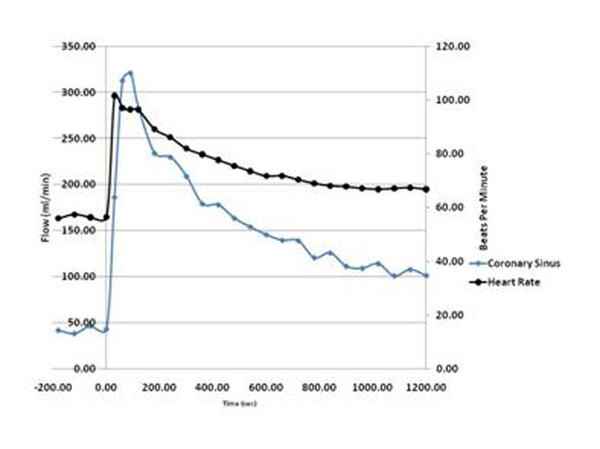
CS Flow vs. HR

**Figure 2:**
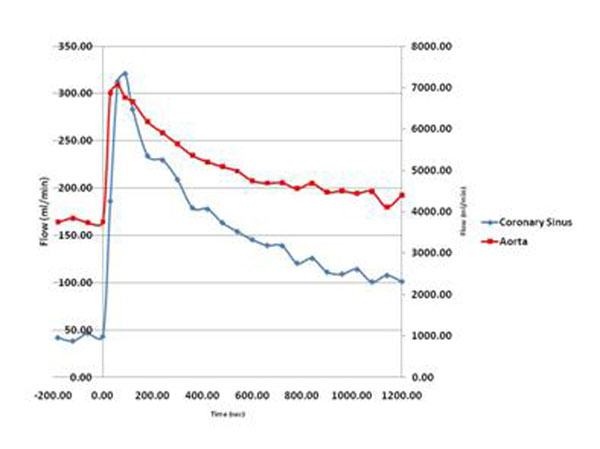
CS Flow vs. Aortic Flow

**Figure 3:**
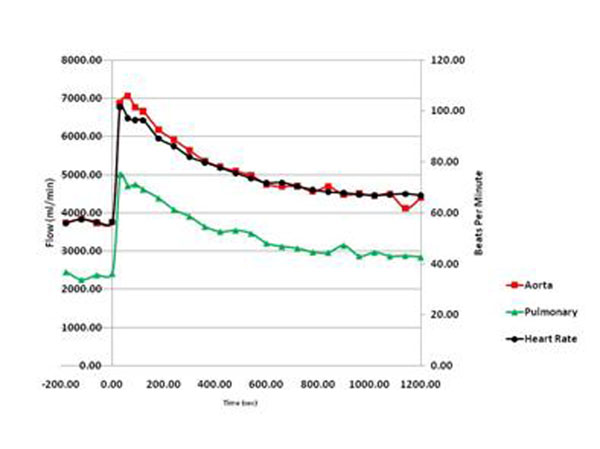
Systemic Flow

**Figure 4:**
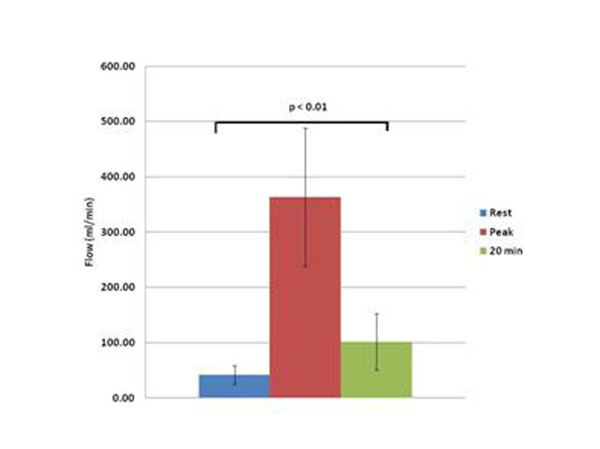
Rest vs. Peak vs. 20 min

Peak CS flow occurred at 101.7 ± 69.1 seconds (median 75 seconds) which was significantly different than the recommended injection time of 20 sec (p<0.001). Flow at 90 seconds was also higher than at 30 seconds (p<0.001). CS flow decreased more rapidly than systemic flow and heart rate however none returned to baseline by 20 min. Figs 1, 2, 3, 4.

## Conclusion

Peak myocardial blood flow occurs later after injection of regadenoson than suggested in the package insert (median 75 or average 102 seconds vs 20 seconds). Optimizing the timing of first-pass perfusion imaging may improve sensitivity in the detection of coronary stenoses.

